# Risk score based on expression of five novel genes predicts survival in soft tissue sarcoma

**DOI:** 10.18632/aging.102847

**Published:** 2020-02-21

**Authors:** Hui-Yun Gu, Chao Zhang, Jia Guo, Min Yang, Hou-Cheng Zhong, Wei Jin, Yang Liu, Li-Ping Gao, Ren-Xiong Wei

**Affiliations:** 1 Department of Spine and Orthopedic Oncology, Zhongnan Hospital of Wuhan University, Wuhan, China; 2 Center for Evidence-Based Medicine and Clinical Research, Taihe Hospital, Hubei University of Medicine, Shiyan, China; 3 Department of Plastic Surgery, Zhongnan Hospital of Wuhan University, Wuhan, China; 4 The Third Clinical School, Hubei University of Medicine, Shiyan, China

**Keywords:** soft tissue sarcoma, least absolute shrinkage and selection operator regression analysis, biomarker, prognostic model, nomogram

## Abstract

In this study, The Cancer Genome Atlas and Genotype-Tissue Expression databases were used to identify potential biomarkers of soft tissue sarcoma (STS) and construct a prognostic model. The model was used to calculate risk scores based on the expression of five key genes, among which MYBL2 and FBN2 were upregulated and TSPAN7, GCSH, and DDX39B were downregulated in STS patients. We also examined gene signatures associated with the key genes and evaluated the model’s clinical utility. The key genes were found to be involved in the cell cycle, DNA replication, and various cancer pathways, and gene alterations were associated with a poor prognosis. According to the prognostic model, risk scores negatively correlated with infiltration of six types of immune cells. Furthermore, age, margin status, presence of metastasis, and risk score were independent prognostic factors for STS patients. A nomogram that incorporated the risk score and other independent prognostic factors accurately predicted survival in STS patients. These findings may help to improve prognostic prediction and aid in the identification of effective treatments for STS patients.

## INTRODUCTION

Soft tissue sarcomas (STS), which are derived from mesenchymal tissues [1], are highly clinically diverse and can originate from many sources, including muscle, adipose tissue, peripheral nerves, blood vessels, and connective tissue [2]. In the United States of America, STS accounts for 1% of cancer cases and 2% of cancer-related deaths [3]. In 2019, 12,750 new STS cases were diagnosed and 5,270 patients died from the disease [4].

Diagnosis and treatment of the multiple histological types of STS are challenging for physicians [5], and a multidisciplinary approach is often beneficial [6]. Because metastasis and disease recurrence are common in STS, patients with localized and early-stage STS could benefit from early diagnosis and radical resection [2, 7–10]. Currently, imaging and biopsy are the primary methods recommended for diagnosing STS. Magnetic resonance imaging is the most effective method for identifying STS originating in the extremities, pelvis, and trunk, while standard radiography and computed tomography are typically used to detect STS in other areas [9]. In the era of precision medicine, identification of biomarkers for and molecular characterization of STS will likely play an increasingly prominent role in diagnosis, treatment, and prognosis prediction. An integrated genomic characterization analysis of 206 adult STS patients conducted by The Cancer Genome Atlas (TCGA) Research Network has been crucial for improving treatment of STS [11]. Nevertheless, identification of additional specific biomarkers would further improve STS treatments.

The TCGA database contains genomic and clinical data from >20,000 primary cancer and matched normal samples representing 33 types of cancer [12]. In addition, the Genotype-Tissue Expression (GTEx) database contains openly available clinical data, including gene expression, quantitative trait locus, and histology images, from 1,000 healthy individuals [13, 14]. In this study, we conducted an integrated analysis of gene expression profiles and clinical data from these databases to identify common gene signatures associated with the development, pathological mechanisms, and prognosis of STS. In addition, we established a prognostic model and nomogram for STS based on clinical data obtained from the TCGA database.

## RESULTS

### Samples and clinical data

TCGA Sarcoma gene expression profiles and clinical data were downloaded from the UCSC Xena Hub datasets. Following data preprocessing, gene expression profiles from 263 STS samples and two matched controls and survival information and other clinical data from 256 patients were included in subsequent analysis. The 263 STS samples included 106 (40%) leiomyosarcomas, 58 (22%) dedifferentiated liposarcomas, 52 (18%) undifferentiated pleomorphic sarcomas, 25 (10%) myxofibrosarcomas, 10 (4%) malignant peripheral nerve sheath tumors, 10 (4%) synovial sarcomas, and two (0.8%) desmoid tumors. Patients ranged from 20 to 89 years in age; 54% were female and 46% were male. The matched controls obtained from the GTEx database included 396 muscle and 515 fat samples.

### Identification and functional annotation of primary differentially expressed genes (DEGs)

**Figure 1:**
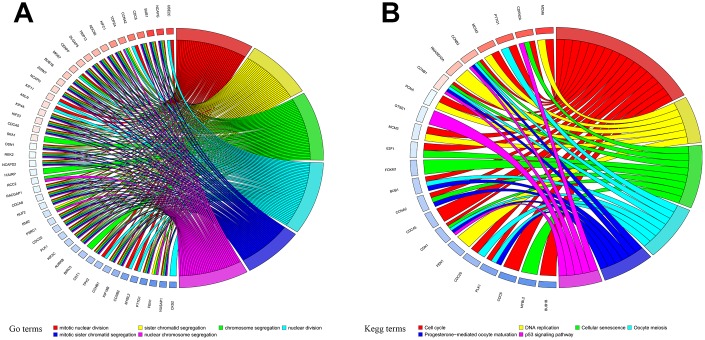
**Functional annotation of primary differentially expressed genes (DEGs).** (**A**) Gene Ontology (GO) functional annotation and (**B**) Kyoto Encyclopedia of Genes and Genomes (KEGG) pathway enrichments for DEGs.

DEGs were obtained from the three groups. A total of 2,290 and 1,301 genes were identified as DEGs in the muscle and fat control groups (|log2 FC|>2, p<0.05), respectively. 775 DEGs were identified in the matched controls group (|log2 FC| >1, p<0.05). Heatmaps of DEGs from the three groups are displayed in Supplementary Figure 1A–1C. 121 DEGs common to all three groups were identified as primary DEGs and examined in subsequent analyses. Functional annotations revealed that primary DEGs were mainly involved in mitotic nuclear division, sister chromatid segregation, chromosome segregation, nuclear division, mitotic sister chromatid segregation, and nuclear chromosome segregation based on the top six terms identified in the GO analysis (p<0.05; Figure 1A, Supplementary Figure 2A, 2B). KEGG analysis showed that primary DEGs were associated with cell cycle, DNA replication, cellular senescence, oocyte meiosis, progesterone-mediated oocyte maturation, and the p53 signaling pathway based on the top six terms (p<0.05; Figure 1B, Supplementary Figure 2C, 2D). The results of these functional annotation suggest that the 121 primary DEGs are associated with the formation and development of tumors.

### Identification of survival-related DEGs and establishment of a prognostic model

A total of 256 patients were randomly assigned to the training (128 patients) and test (128 patients) groups. A total of 44 DEGs were selected via univariate Cox regression analysis (p<0.05). Following Lasso regression analysis, seven DEGs were selected for multivariate Cox regression analysis (optimal log(Lambda): 7; Figure 2A, 2B). Finally, five key genes highly associated with survival were used to establish the prognostic model: risk score = −0.201×exp(TSPAN7) + 0.284×exp(MYBL2) + 0.941×exp(GCSH) + 0.159×exp(FBN2) + 0.417×exp(DDX39B) (Table 1). Patients were separated into high- and low-risk groups based on this risk score; those in the high-risk group had lower survival rates (p=6.691e−05, Figure 3A). As shown in Figure 3D, these five survival-related DEGs performed satisfactorily in predicting prognosis of STS patients (area under the curve: 0.781). There were obvious differences in expression of the key genes between patients with lower and higher risk scores (Figure 3G). Moreover, those with higher risk scores had shorter survival times. Similar results were obtained for the test group, which validated the prognostic model (Figure 3B, 3E, 3H). Finally, we confirmed the value of this prognostic model by testing it in the overall patient group, which included all patients in the study (Figure 3C, 3F, 3I).

**Figure 2:**
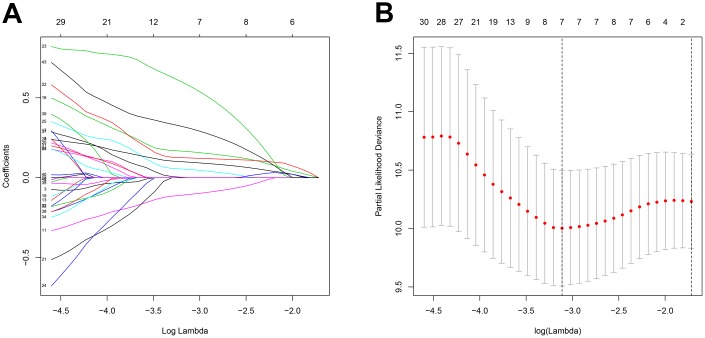
**Feature selection using the Lasso regression model.** (**A**) Lasso regression analysis coefficients. (**B**) Selection of tuning parameters in the Lasso regression analysis based on 1,000 cross-validations.

**Table 1:** Multivariate Cox regression analysis of key genes.

**Genes**	**Overall survival**				
	**coef**	**HR**	**95% CI**		**p value**
TSPAN7	-0.201	0.818	0.652	1.026	0.082
MYBL2	0.284	1.328	1.057	1.669	0.015
GCSH	0.941	2.562	1.388	4.728	0.003
FBN2	0.159	1.172	1.006	1.366	0.041
DDX39B	0.417	1.518	1.071	2.150	0.019

Note: coef: coefficient; HR: hazard ratio; CI: confidence interval.

**Figure 3:**
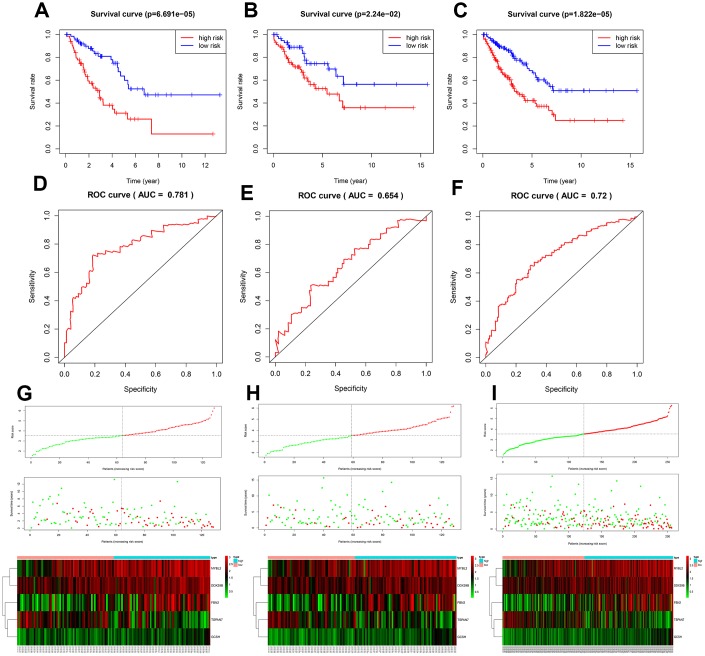
**Assessment of the prognostic model.** Survival analyses for the training (**A**), test (**B**), and overall (**C**) datasets. Receiver operating curves (ROC) of the prognostic model in the training (**D**), test (**E**), and overall (**F**) datasets. Differences in risk score, survival time, and gene expression between the high- and low-risk groups in the training (**G**), test (**H**), and overall (**I**) datasets.

### Expression levels of the five key DEGs in STS patients

Among the five key genes, expression of MYBL2 and FBN2 was increased, and expression of TSPAN7, GCSH, and DDX39B was decreased, in STS patients (p<0.001; Supplementary Figure 3A and 3B). Expression levels of all of these key genes except DDX39B, which was not indexed in the external dataset (GSE21122), were validated in Supplementary Figure 3C. The five key genes effectively discriminated between STS patients and controls (Supplementary Figure 4). As shown in Supplementary Figure 5A and 5B, the five key genes could also distinguish between low- and high-risk patients and healthy individuals, further validating the accuracy of the prognostic model (p<0.001). Moreover, there were significant differences in expression of the five key genes between all histological types and controls (Supplementary Figure 6A and 6B). This result was also validated using the GSE21122 datasets (Supplementary Figure 6C), indicating these five key genes were suitable for predicting prognosis in all histological types included in this study.

### Alterations in key genes in STS

**Figure 4:**
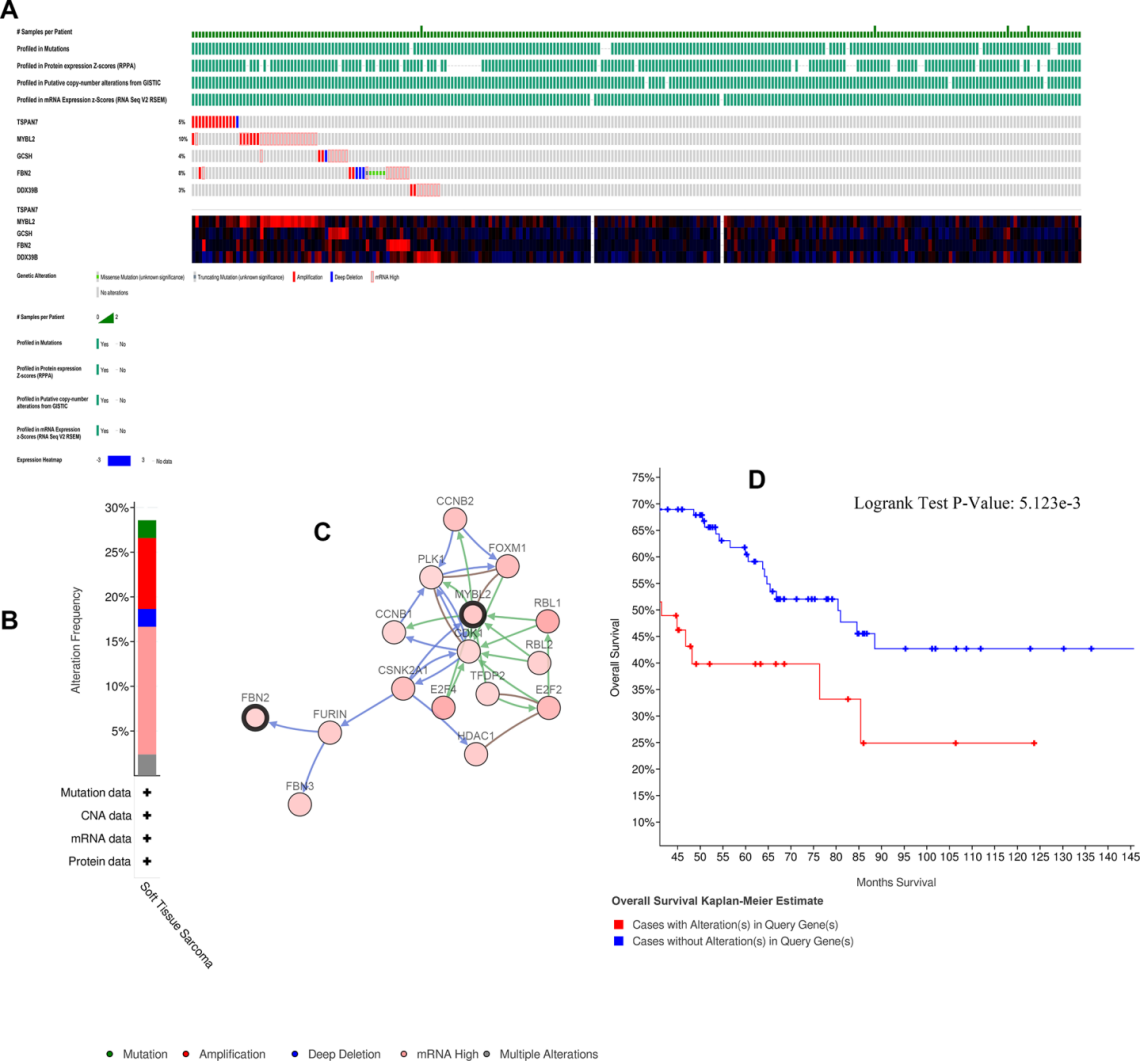
**Alterations in expression of the five key genes.** (**A**) 73 of 265 samples (28%) had alterations of the five key genes. (**B**) Frequencies of different alterations. (**C**) Network of key genes and most frequently altered neighbor genes. (**D**) Survival analysis for patients with and without alterations in the five key genes.

Alterations of the key genes were explored using data obtained from the cBioPortal database. Of the 265 samples included, 73 (28%) had alterations in the key genes (Figures 4A, 4B); the network of key genes and most frequently altered neighbor genes is displayed in Figure 4C. Furthermore, patients with alterations in the key genes had shorter OS (Figure 4D).

### Relationships between immune infiltration, risk scores, and gene expression

**Figure 5:**
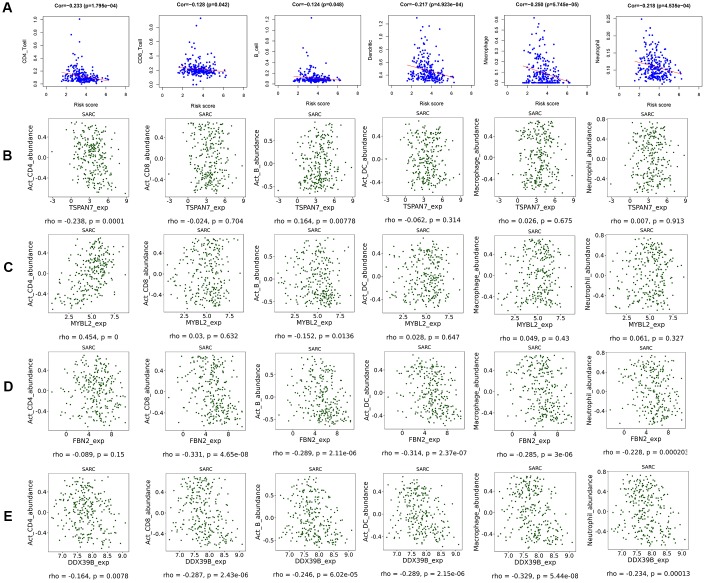
**Scatter diagram of the relationship between immune cell infiltration, risk scores, and key gene expression.** (**A**) Relationships between immune cell infiltration and risk scores. (**B**) Relationships between immune cell infiltration and expression of the TSPAN7 (**B**), MYBL2 (**C**), FBN2 (**D**), and DDX39 (**E**) genes.

CD4+ T cell (correlation (cor)= -0.233, p=1.795e-04), CD8+ T cell (cor= -0.128, p=0.042), B cell (cor= -0.124, p=0.048), dendritic cell (cor= -0.217, p=4.923e-04), macrophage (cor= -0.250, p=5.745e-05), and neutrophil (cor= -0.218, p=4.535e-04) infiltration were negatively correlated with STS patient risk scores (Figure 5A). Similar negative correlations were observed between immune cells and FBN2 and DDX39B expression specifically (Figure 5D, 5E). TSPAN7 expression was negatively correlated with CD4+ T cell infiltration, but positively correlated with B-cell infiltration (Figure 5B). MYBL2 expression was positively correlated with CD4+ T cell infiltration, but negatively correlated with B-cell infiltration (Figure 5C). Moreover, low-risk patients had higher expression of immune checkpoint molecules PDCD1 and CD274 (Supplementary Figure 7). No differences in CTLA4 and LAG3 expression were observed between high- and low-risk patients (Supplementary Figure 7).

### GSEA analysis for the five key genes

The top pathways for each key gene are illustrated in Supplementary Figure 8A–8E; overall, they were highly associated with cell cycle and cancer pathways. These results further validate the crucial roles these genes play in STS and tumors in general.

### Identification of prognostic factors and nomogram construction

**Figure 6:**
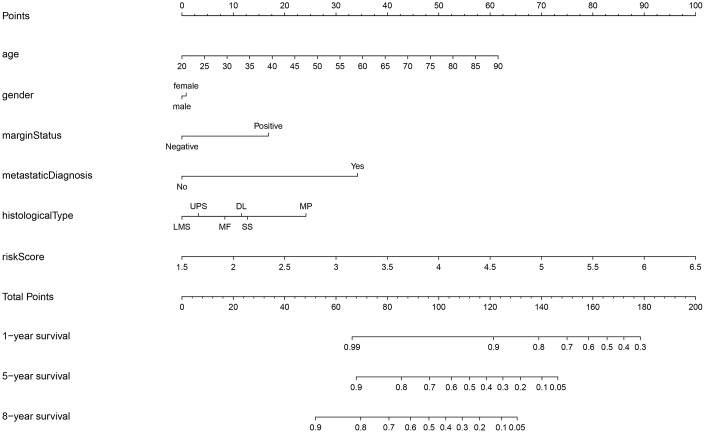
**Nomogram for STS.** STS: soft tissue sarcoma; LMS: leiomyosarcomas; UPS: undifferentiated pleomorphic sarcoma; MF: myxofibrosarcomas; DL: dedifferentiated liposarcomas; SS: synovial sarcomas; MP: malignant peripheral nerve sheath tumors.

Univariate and multivariate Cox analyses indicated that age, margin status, presence of metastasis, and risk score had significant impacts on prognosis (Table 2). In addition to these four factors, sex and histological type were used to construct the nomogram for STS patients displayed in Figure 6. Internal validation showed that the predictive accuracy for STS as calculated using the C-index was 0.782. Actual survival rates and predictions obtained using the nomogram were largely concordant (Supplementary Figure 9A–9C).

**Table 2:** Univariate and multivariate analysis of clinical factor and risk score.

**Variables**	**Univariate analysis**					**Multivariate analysis**			
	**HR**	**95% CI**		**P value**		**HR**	**95% CI**		**P value**
Age	1.023	1.007	1.040	0.004		1.034	1.015	1.053	2.865E-04
Gender	0.853	0.571	1.275	0.438		NA	NA	NA	NA
Race	1.257	0.591	2.671	0.553		NA	NA	NA	NA
Sample weight	1.000	0.999	1.000	0.295		NA	NA	NA	NA
Total necrosis percent	1.208	0.953	1.532	0.119		NA	NA	NA	NA
Margin status	1.899	1.175	3.070	0.009		2.155	1.262	3.679	0.005
Metastatic diagnosis	2.939	1.788	4.831	2.123E-05		3.937	2.252	6.884	1.533E-06
Radiation therapy	0.604	0.280	1.301	0.198		NA	NA	NA	NA
Histological type	0.972	0.865	1.093	0.636		NA	NA	NA	NA
Risk score	1.908	1.506	2.417	8.518E-08		2.355	1.710	3.243	1.554E-07

Note: HR: hazard ratio; CI: confidence interval; NA: not available.

### Identification of small-molecule drugs

The 107 upregulated and 14 downregulated DEGs were mapped into the Connectivity Map database, which was then used to identify the top 10 small-molecule drugs most likely to be effective based on p-values < 0.05 (Table 3). Camptothecin, daunorubicin, 0175029-0000, resveratrol, and trichostatin A were the top molecules likely to act on the gene targets obtained in our comparison of tumor and normal tissues; these drugs might therefore be particularly useful for treating STS.

**Table 3:** Potential small-molecule drugs identified using the Connectivity Map database.

**Rank**	**Cmap name**	**Mean**	**n**	**Enrichment**	**P**	**Specificity**	**Percent non-null**
1	camptothecin	-0.727	3	-0.985	0	0.0568	100
2	daunorubicin	-0.661	4	-0.952	0	0.0088	100
3	0175029-0000	-0.753	6	-0.952	0	0	100
4	resveratrol	-0.726	9	-0.836	0	0	100
5	trichostatin A	-0.419	182	-0.394	0	0.3663	84
6	GW-8510	-0.663	4	-0.927	0.00002	0.0916	100
7	colistin	0.567	4	0.873	0.00036	0	100
8	dipyridamole	-0.506	6	-0.756	0.0004	0.0123	100
9	fludrocortisone	0.269	8	0.669	0.00048	0.0177	50
10	apigenin	-0.619	4	-0.873	0.00056	0.0163	100

**Note:** camp: Connectivity Map.

### DISCUSSION

Because it encompasses a large and heterogenous group of malignant tumors with diverse origins, STS is difficult to diagnosis and treat effectively. In addition to conventional diagnosis and treatment, identification of novel specific biomarkers might improve outcomes for both early and advanced stage STS patients [15]. In this study, we attempted to identify potential biomarkers for risk stratification and prognosis prediction in STS patients, as well as small-molecule drugs that might aid in treatment by targeting these biomarkers.

The TCGA and the GTEx databases were utilized to identify differentially expressed genes (DEGs) that might serve as potential biomarkers for STS; univariate Cox regression analysis, Lasso regression analysis, and multivariate Cox regression analysis were then used to select key genes for inclusion in the prognostic model. The DEGs of interest were mainly enriched in mitotic nuclear division, sister chromatid segregation, chromosome segregation, and nuclear division, which are highly associated with tumorigenesis. They were also involved in cell cycle, DNA replication, and the p53 signaling pathway. The p53 signaling pathway is a well-established pathway associated with various types of cancer [16], including sarcomas [17]. A prognostic model was used to generate risk scores based on expression of the five key genes, among which MYBL2 and FBN2 were upregulated, while TSPAN7, GCSH, and DDX39B were downregulated, in STS; validation studies confirmed that this risk score was highly associated with survival. The model was effective in distinguishing between high- and low-risk STS patients and between patients and healthy individuals, regardless of the histological type.

Changes in expression of the five key genes identified here have been reported in different types of cancer, including lung [18], renal [19], breast [20], colorectal [21], prostate [22], and multiple myeloma [23]. In an analysis of gene expression profiles from 13 primary and 15 metastatic uterine leiomyosarcoma cases, Davidson et al. [24] reported that TSPAN7 was overexpressed in primary uterine leiomyosarcoma; however, in our study, TSPAN7 was downregulated in all histological types of STS. This inconsistency might be due to the small sample size in the Davidson study. Consistent with our results, increased expression of TSPAN7, which can inhibit the development of multiple myeloma *in* *vivo* [23], was associated with longer survival time in clear-cell renal cell carcinoma [25]. FBN2 has been identified as a diagnostic biomarker in leiomyosarcoma and rhabdomyosarcoma [26, 27]. Additionally, aberrant methylation of FBN2 has been observed in breast cancer, non-small cell lung cancer, and esophageal squamous cell carcinoma [28–30]; FBN2 methylation might negatively impact STS prognosis as well. To our knowledge, the role of GCSH has not been examined in STS, but only in breast cancer and papillary thyroid cancer [20, 31]. MYBL2 is associated with poor prognosis in numerous cancers and plays a vital role in the regulation of cell proliferation, cell survival, and differentiation [32]. For example, MYBL2 was recently found to promote progression of Ewing sarcoma [33]. Here, overexpression of MYBL2 was associated with poor outcomes in STS patients. DDX39B, a DExD RNA helicase, is involved in pre-mRNA splicing and nuclear export of mRNAs [34]. Awasthi et al. [35] found that DDX39B could promote global translation and cell proliferation through upregulation of pre-ribosomal RNA, ultimately leading to oncogenesis. In addition, DDX39B is a crucial contributor to Kaposi's sarcoma-associated herpesvirus intronless mRNA nuclear export and virus replication [36]. Because all histological types of STS were characterized by changes in the expression of these five key genes, they might be particularly useful as new prognostic biomarkers for STS. However, the specific roles of these genes in STS need to be examined in future studies.

In this study, we performed multilevel analyses to further explore associations between key genes in STS and immune infiltration, gene alterations, and GSEA pathways. Negative correlations between infiltration of six types of immune cells and risk scores indicated that increased immune cell infiltration contributed to better survival in STS, which is consistent with previous studies [11, 37]. The TCGA Research Network [11] reported that higher NK, T, and dendritic cell levels were associated with better outcomes. In contrast to our findings, Koirala et al. [38] found that increased dendritic cell (DC) and macrophage levels negatively impacted survival in human osteosarcoma. The absence of lymphatic vessels, and the resulting inhibition of antigen-presenting capacity, in human bone tissue might explain these detrimental effects of DCs [39]; this might also highlight important differences in immune infiltration between STS types containing lymphatic vessels and osteosarcoma. Conflicting results have been obtained regarding the association between macrophage infiltration and osteosarcoma prognosis [40], and additional studies are needed on this topic. In this study, we found that expression of two of the key genes, DDX39B and FBN2, was negatively correlated with infiltration of most immune cell types. MYBL2 expression was positively correlated with CD4+ T-cell infiltration, but negatively correlated with B-cell infiltration. TSPAN7 expression was negatively correlated with CD4+ T-cell infiltration and positively with B-cell infiltration. Finally, GCSH expression was not correlated with infiltration for any of the immune cell types examined. We also demonstrated that expression of PDCD1 and CD274 was higher in low-risk patients, suggesting that our prognostic model could potentially identify patients who would benefit from treatment with immune checkpoint inhibitors. Our use of five key genes together in a single model improved its prognostic value compared to the individual genes, based on the comparisons of risk scores with the individual gene expression in the correlations with immune cell infiltration. In addition, survival times were substantially reduced in patients with alterations in these key genes, indicating that they possess accurate prognostic power. Finally, a GSEA analysis revealed that the key genes promoted cell proliferation as well as cancer development and progression via different cell cycle, DNA replication, mismatch repair, and cancer-associated pathways (e.g., phosphatidylinositol signaling system [41], basal cell carcinoma, transforming growth factor beta signaling pathway [42], WNT signaling pathway [43], and the p53 signaling pathway). These signaling pathways have also been reported as important regulators in osteosarcoma and STS [43, 44]. Finally, these key genes might also affect development and progression of STS through interactions with gene fusion products and miRNAs, which not only play important regulatory roles but can also act as therapeutic targets in sarcoma [45]. For example, EWSR1 fusion is common in Ewing sarcoma [46], and EWSR1-FLI1 regulates the expression of MYBL2 [33]. Additional studies of such mechanisms might also help improve diagnosis and treatment of STS.

Our prognostic model based on five key genes was able to stratify STS patients into clinically meaningful high- and low-risk groups which differed significantly in terms of survival. The accuracy of the prognostic model was validated using the test group. In addition, univariate and multivariate Cox regression analyses of our model demonstrated that age, margin status, diagnosis of metastasis, and risk score were independent prognostic factors for STS. Prognostic predictions for STS patients are currently based on the presence of metastases as well as tumor grade, size, and depth [47, 48]. While previous studies have constructed nomograms for prognostic predictions in STS [49], gene expression is not typically included. In this study, we incorporated our risk score together with multiple clinical factors (e.g., age, sex, margin status, diagnosis of metastasis, histological type) to generate a prognostic nomogram with high predictive accuracy for STS patients.

Finally, we identified small-molecule drugs that might improve STS treatment by targeting the key genes using the Camp database. One such drug, Camptothecin, is a topoisomerase inhibitor [50] that inhibited cell proliferation and showed anticancer activity in colon, lung, ovarian, breast, liver, pancreas, and stomach cancer [51] and in Ewing sarcoma [52]. Furthermore, combinations of immune checkpoint inhibitors and the small-molecule drugs might also help improve treatments for STS [53].

This integrated analysis of multiple databases enhanced the robustness and reproducibility of our conclusions; the inclusion of information from the GTEx database was especially helpful in compensating for the paucity of control samples in the TCGA database. In addition, our inclusion of both gene expression and clinical data might render our results especially applicable to STS patients in the clinical setting. However, analysis of additional clinical data is necessary to confirm these results.

## CONCLUSION

This study demonstrated that five key genes (i.e., MYBL2, FBN2, DDX39B, TSPAN7, and GCSH) highly associated with prognosis, and immune infiltration, could promote STS via different signaling pathways. The prognostic model based on these five key genes demonstrated excellent performance in terms of risk stratification of patients and prediction of survival. Furthermore, the nomogram integrating multiple genes and clinical factors could provide specific predictions for the survival of individuals with STS. Based on the combination of gene and clinical data, this study may contribute to the management of STS.

## MATERIALS AND METHODS

### Data collection and preprocessing

Gene expression profile and clinical data from the TCGA and GTEx databases were downloaded from the University Of California Santa Cruz (UCSC) Xena Hub datasets (https://xenabrowser.net/). Gene expression data were downloaded from TCGA Sarcoma, which were obtained using the Illumina HiSeq 2000 RNA Sequencing platform by the University of North Carolina (Chapel Hill, NC, USA) TCGA genome characterization center. Data were obtained for 265 samples (263 samples with STS and two matched controls) and were log2(x+1) transformed. We also downloaded gene expression profiles for muscle and fat tissues, which were the two most common histological types among TCGA samples, from the GTEx database for use as additional matched controls. GTEx gene expression data were also log2(x+1) transformed to allow comparisons to TCGA data. Transformation of Ensembl identifiers and normalization of expression between the TCGA and GTEx datasets were also performed prior to subsequent differential analysis. GSE21122 gene expression profiles [54] for 149 STS samples and nine normal fat tissue samples were downloaded from the Gene Expression Omnibus database (https://www.ncbi.nlm.nih.gov/geo/) and used as the validation dataset. If a database did not include an STS category, sarcoma sample data was used as STS data; most databases include only STS and not bone tumors in the sarcoma category.

### Identification of differentially expressed genes (DEGs)

The DEG analyses were performed using the “limma” package [55] in R statistical software, version 3.53. DEGs were divided among three groups: “tumors” vs. “muscle tissues” + “matched controls” from TCGA Sarcomas (hereinafter “muscle controls”), “tumors” vs. “fat tissues” + “matched controls” (hereinafter “fat controls”), and “tumors” vs. “matched controls”. DEGs were defined by |log2 FC|>2 and p<0.05 in the first two groups. Because the third group included only two matched controls, DEGs in that group were defined by |log2 FC|>1 and p<0.05 to increase the number of identified genes. Only DEGs that were identified in all three groups were included in subsequent analyses.

### Functional annotation of DEGs

Gene Ontology (GO) functional annotation and Kyoto Encyclopedia of Genes and Genomes (KEGG) pathway enrichment were performed in R using the “clusterProfiler” package [56]. Adjusted p<0.05 was considered statistically significant in the GO and KEGG analyses. Bar plots, dot plots, and chord plots were constructed to visualize functional annotation results via the “GOplot” package [57] in R.

### Identification of survival-related DEGs and establishment of a prognostic model

All available samples were randomly assigned to the training and test datasets. The training group was used to establish the prognostic model, which was then validated using the test group. First, the primary DEGs identified above were used in a univariate Cox regression analysis in the training dataset using the “survival” package in R. DEGs with p<0.05 in the univariate Cox regression analysis were then selected for least absolute shrinkage and selection operator (Lasso) regression analysis using the “glmnet” and “survival” packages [58]; Lasso regression analysis, which detects the optimal lambda value based on 1,000 cross-validations, is particularly useful in high-dimension datasets [59]. The DEGs of interest identified in the Lasso regression analysis were then subjected to multivariate Cox regression analysis. The prognostic model was constructed based on the following equation: $\text{risk}\;\text{score}=\sum_{i=1}^{n}\beta \text{i}\times \exp({\text{G}}_{\text{i}})$; where *n* is the number of genes identified for the multivariate Cox regression model; *exp(G_i_)* is the expression value of gene *i*; and *β_i_* refers to the coefficient for gene *i*. The DEGs included in the prognostic model were regarded as key genes associated with survival. Patients were divided into “high-risk” and “low-risk” groups based on the median risk score obtained using this prognostic model. To confirm the results, the prognostic model was also used to analyze the test datasets. Finally, analyses of overall survival (OS) were performed to evaluate differences in OS between high- and low-risk patients in the training and test datasets. The receiver operating characteristic (ROC) curve was plotted using the “survivalROC” package to determine the specificity and sensitivity of the risk model. We also analyzed OS and plotted ROC curves when all samples included in the study were combined in a single dataset.

### Expression of the key genes in STS

Differences in expression of the key genes between the STS and normal groups and between the high-risk, low-risk, and normal groups were compared using the “ggstatsplot” package (http://doi.org/10.5281/zenodo.2074621); ROC curves for the five key genes were plotted for each group. Data from the TCGA and GTEx databases and the GSE21122 dataset were then used to investigate differences in expression of the key genes between various histological types and matched controls.

### Alterations in the key genes in STS

The cBio Cancer Genomics Portal database (cBioPortal database), (http://cbioportal.org) containing multidimensional cancer genomics data sets from >5,000 tumor samples enables multilevel analysis for a diverse set of tumors [60]. This database was used to investigate associations between alterations in the key genes and survival in STS patients.

### Immune infiltration analysis

Immune infiltration analysis was performed using the Tumor Immune Estimation Resource (TIMER) database, which includes molecular characterizations of six tumor-infiltrating immune subsets in 32 types of cancer (https://cistrome.shinyapps.io/timer/) [61, 62]. A matrix of six immune cell types (CD4+ T cells, CD8+ T cells, B cells, dendritic cells, macrophages and neutrophils) from the TIMER database estimation module were downloaded and used to explore the relationship between immune cells and risk scores. We also investigated associations between the five key genes and immune cells in TISIDB, an integrated repository portal for tumor–immune system interactions [63], to understand the impact of gene expression on the immune infiltration. Accumulating evidence has demonstrated that patients’ responses to immune checkpoint inhibitors are associated with the expression level of immune checkpoint molecules and immune cell infiltration [38, 64, 65]. Differences in the common immune checkpoint molecules PD-L1(CD274), PD1(PDCD1), CTLA4, and LAG3 between high-risk and low-risk patients were examined to determine whether the five key genes might be associated with immune checkpoint inhibitor efficacy. A p<0.05 denoted statistical significance.

### Gene Set Enrichment Analysis (GSEA) of the five key genes

Gene expression profiles for the 263 STS patients from the TCGA database were utilized for GSEA using GSEA 3.0 software [66]. Patients were divided into the high- and low-expression groups based on the median of key gene expression. KEGG pathways for the key genes were determined based on the p<0.05 or a false discovery rate <0.05, and the top terms were visualized using R.

### Identification of the prognosis-related clinical factors

Univariate and multivariate Cox regression analyses were performed to explore the impact of clinical factors on the prognostic model. Because clinical data were not available for all samples, the training and test datasets were combined and analyzes as a single dataset in subsequent analyses. The following factors were included in the univariate Cox regression analysis: age, sex, race, sample weight, percent of total necrosis, margin status, diagnosis of metastasis, radiation therapy, histological type, and risk score. Factors identified as significant in that analysis were used in multivariate Cox regression analyses to identify independent prognostic factors. These analyses were conducted using the “survival” package.

### Construction of the nomogram and internal validation

We used R to plot the nomogram for OS in STS patients. The nomogram incorporated sex, histological type, and the independent prognostic factors identified in multivariate Cox regression analysis. Bootstrap resampling was used to internally validate the nomogram based on concordance index (C-index) values, which were obtained through 1,000 resampling events, and calibration curves, which were plotted to evaluate concordance between actual and predicted survival rates [67].

### Identification of small-molecule drugs

The Connectivity Map database contains expression data from cultured human cells treated with bioactive small molecules. These data can be used to explore associations between small-molecule drugs and genes of interest identified in patients, to identify potential mechanisms of action for these drugs, and to promote novel drug design [68]. Small-molecule drugs that might be useful for the treatment of STS were identified by mapping the primary upregulated and downregulated DEGs into the database.

## Supplementary Material

Supplementary Figures
