# Supplementary material for: Risk score based on expression of five novel genes predicts survival in soft tissue sarcoma
Source: Aging (Albany NY). 2020 Feb 21;12(4):3807–27. doi: 10.18632/aging.102847 (PMC7066896; doi:10.18632/aging.102847)
Supplement: Supplementary Figures [file aging-12-102847-s001..pdf]

SUPPLEMENTARY FIGURES

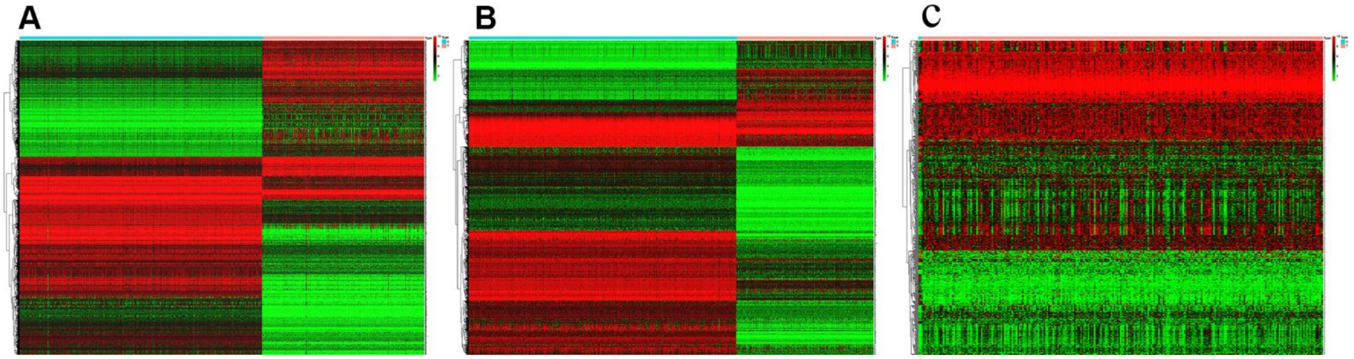

Supplementary Figure 1. Heatmaps of DEGs. Heatmaps of DEGs from “tumors” vs. “muscle controls” (**A**), “tumors” vs. “fat controls” (**B**), and “tumors” vs. “matched controls” (**C**).

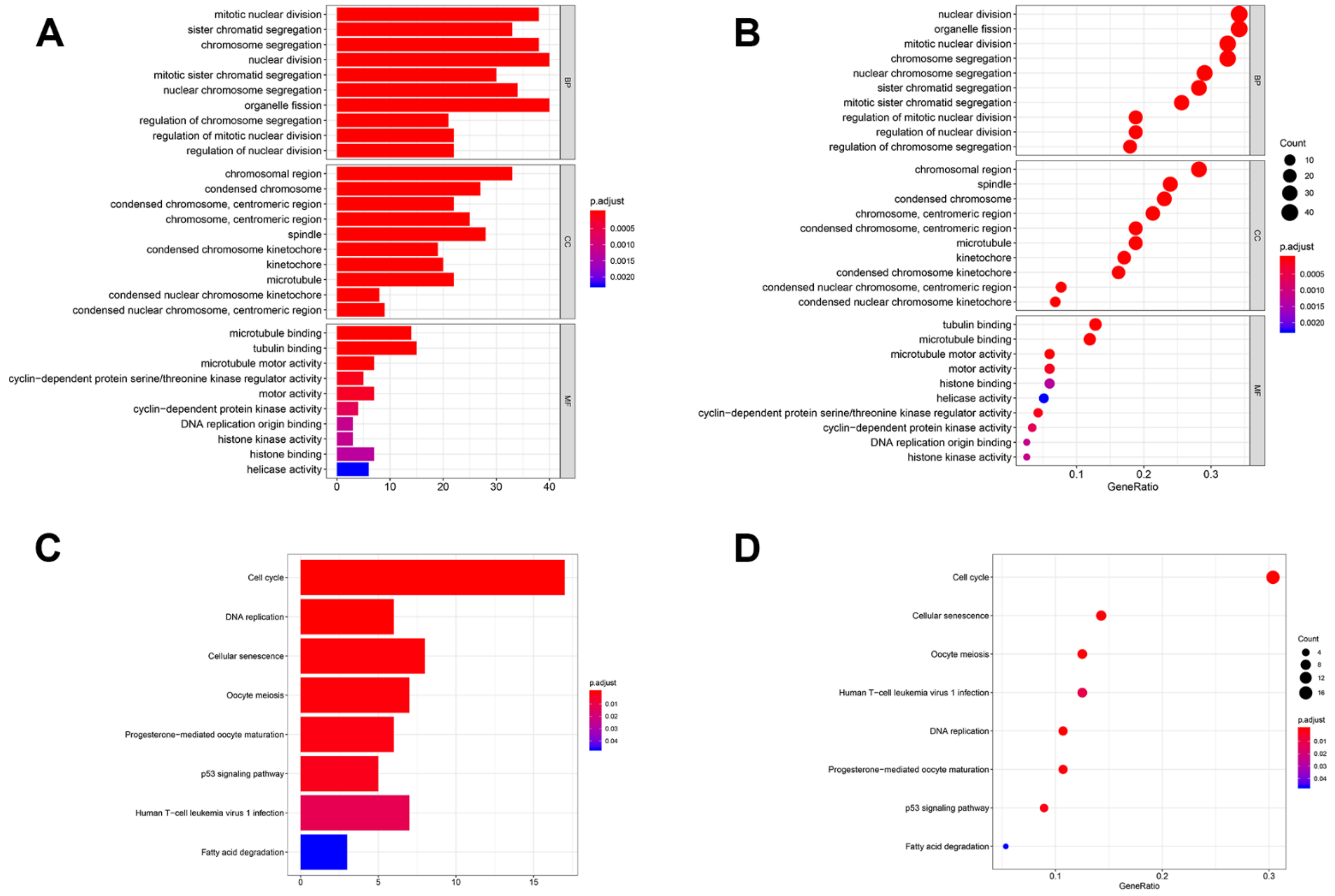

Supplementary Figure 2. Functional annotation of primary differentially expressed genes (DEGs). The Gene Ontology (GO) functional annotation (**A** and **B**) and Kyoto Encyclopedia of Genes and Genomes (KEGG) pathway enrichments (**C** and **D**) of DEGs.

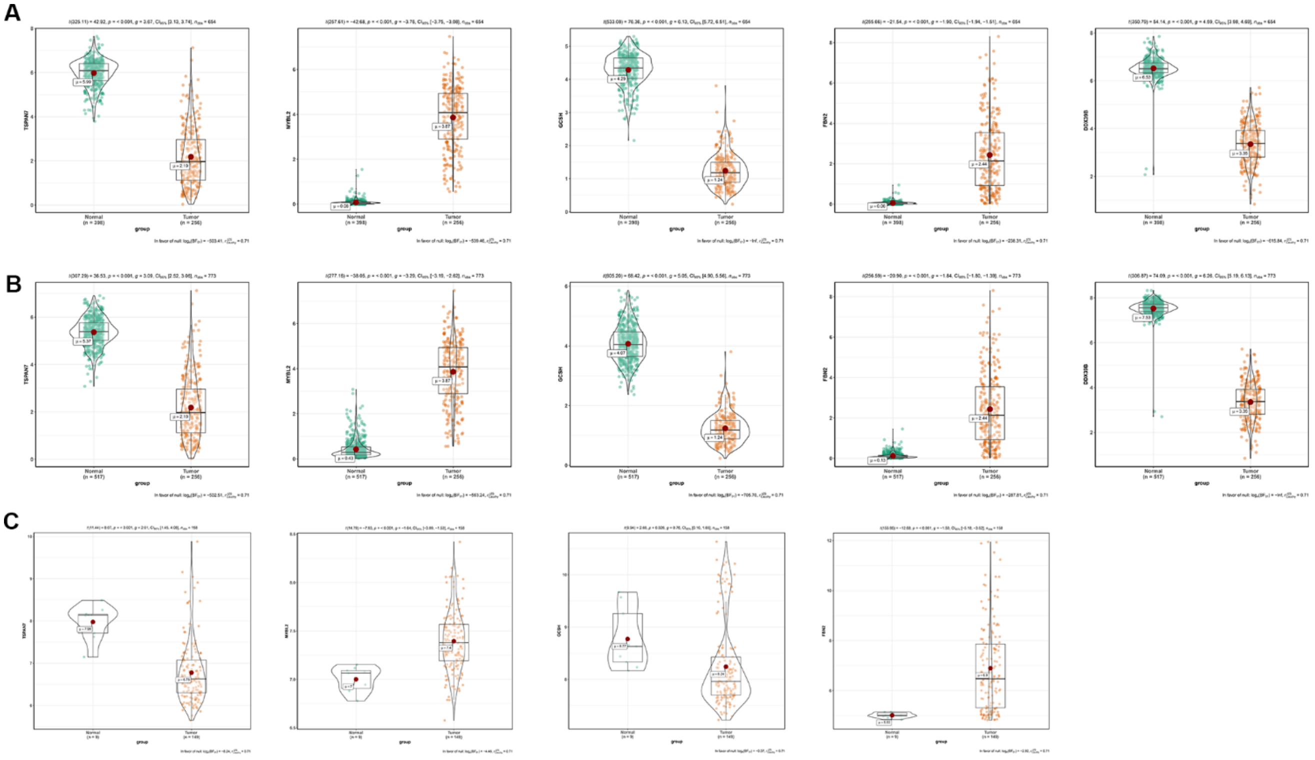

Supplementary Figure 3. Differences in the expression of key genes between soft tissue sarcoma (STS) and normal groups. (**A**) Differences in the expression of key genes between STS and muscle controls. (**B**) Differences in the expression of key genes between STS and fat controls. (**C**) Differences in the expression of five key genes between STS and normal groups in the GSE21122 datasets.

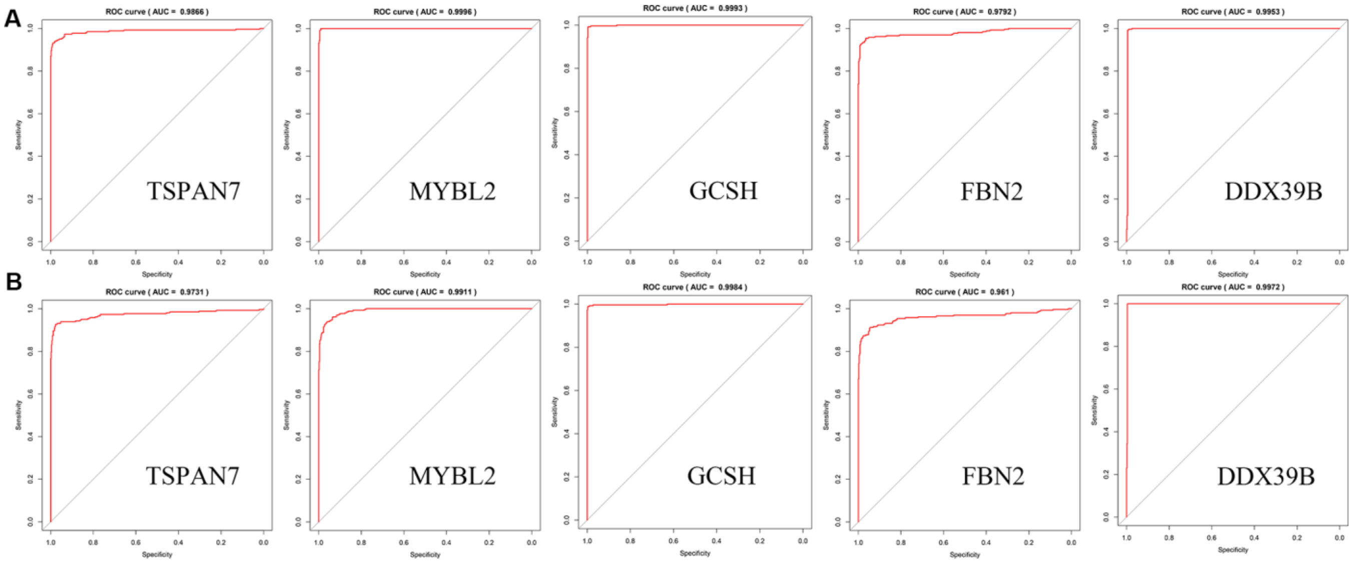

Supplementary Figure 4. Discrimination ability of key genes between STS and controls. (**A**) Discrimination ability of the five key genes between STS and muscle controls. (**B**) Discrimination ability of the five key genes between STS and fat controls.

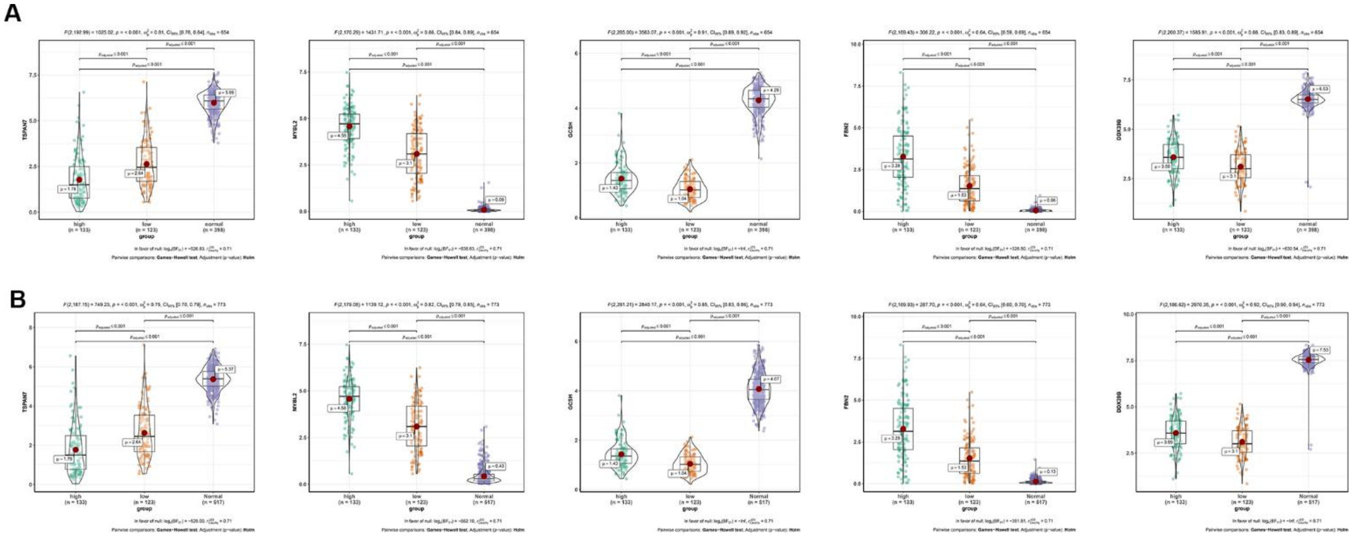

Supplementary Figure 5. Differences in the expression of key genes among high-risk and low-risk patients, and healthy (control) individuals. (**A**) Differences in the expression of key genes among high-risk and low-risk patients, and healthy (muscle controls) individuals. (**B**) Differences in the expression of key genes among high-risk and low-risk patients, and healthy (fat controls) individuals.

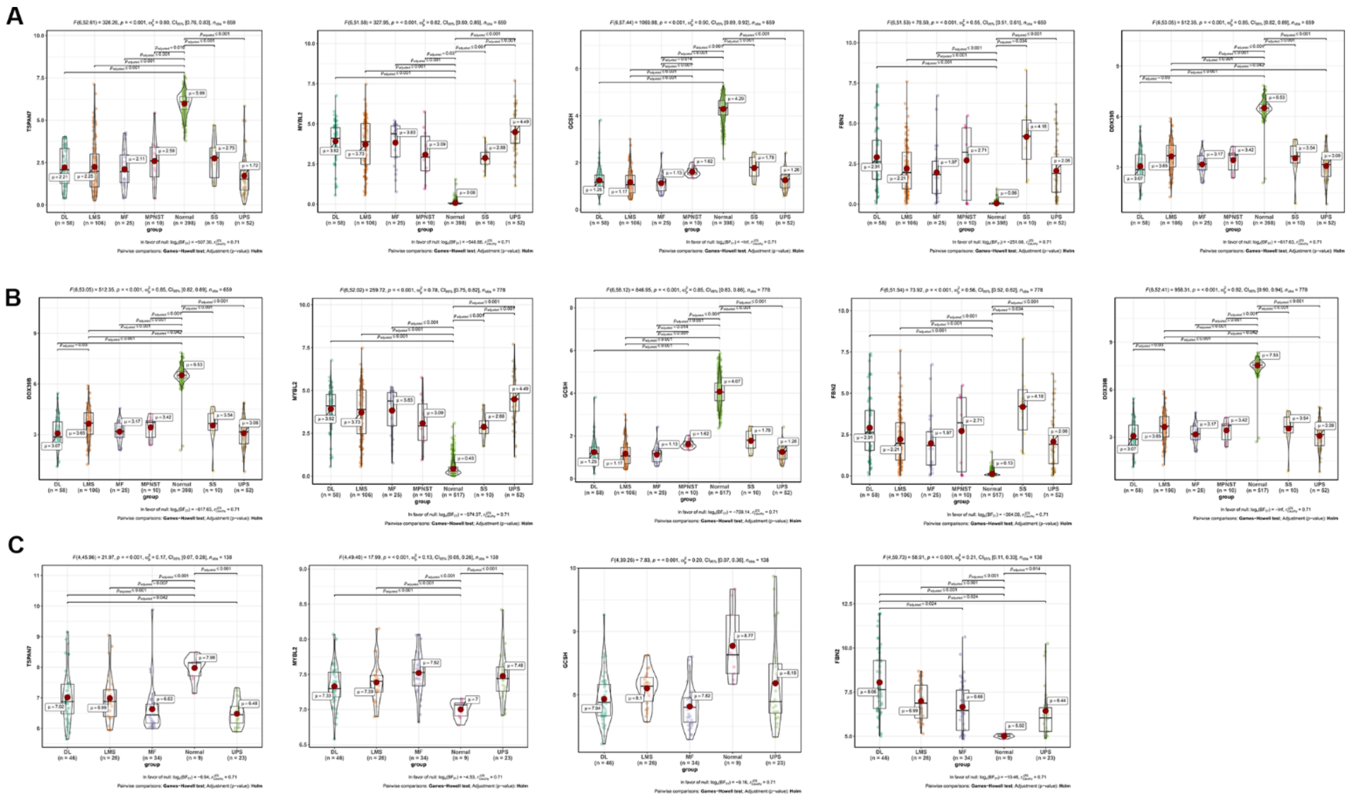

Supplementary Figure 6. Differences in the expression of key genes between all histological types and normal controls. (**A**) Differences in the expression of key genes between all histological types and muscle controls. (**B**) Differences in the expression of key genes between all histological types and fat controls. (**C**) Differences in the expression of key genes between all histological types and normal controls in the GSE21122 datasets.

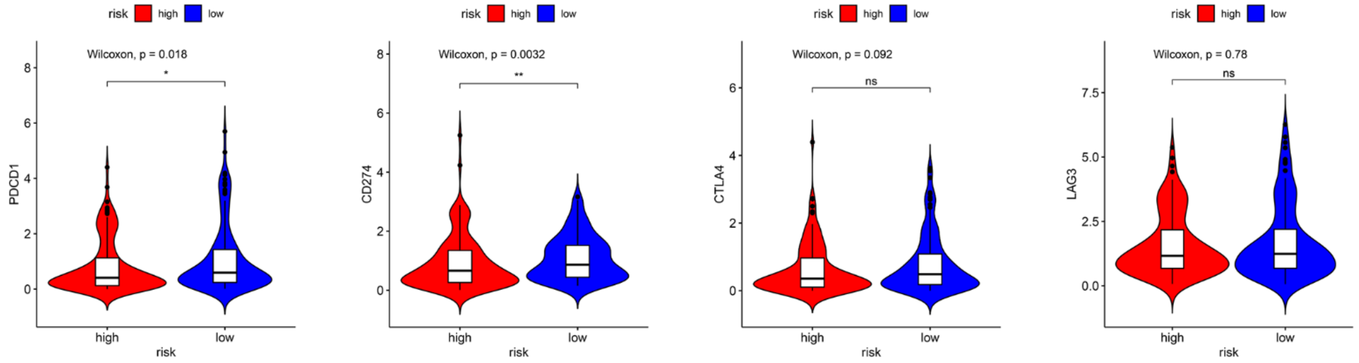

Supplementary Figure 7. Differences in the common immune checkpoint molecules between high-risk and low-risk patients.

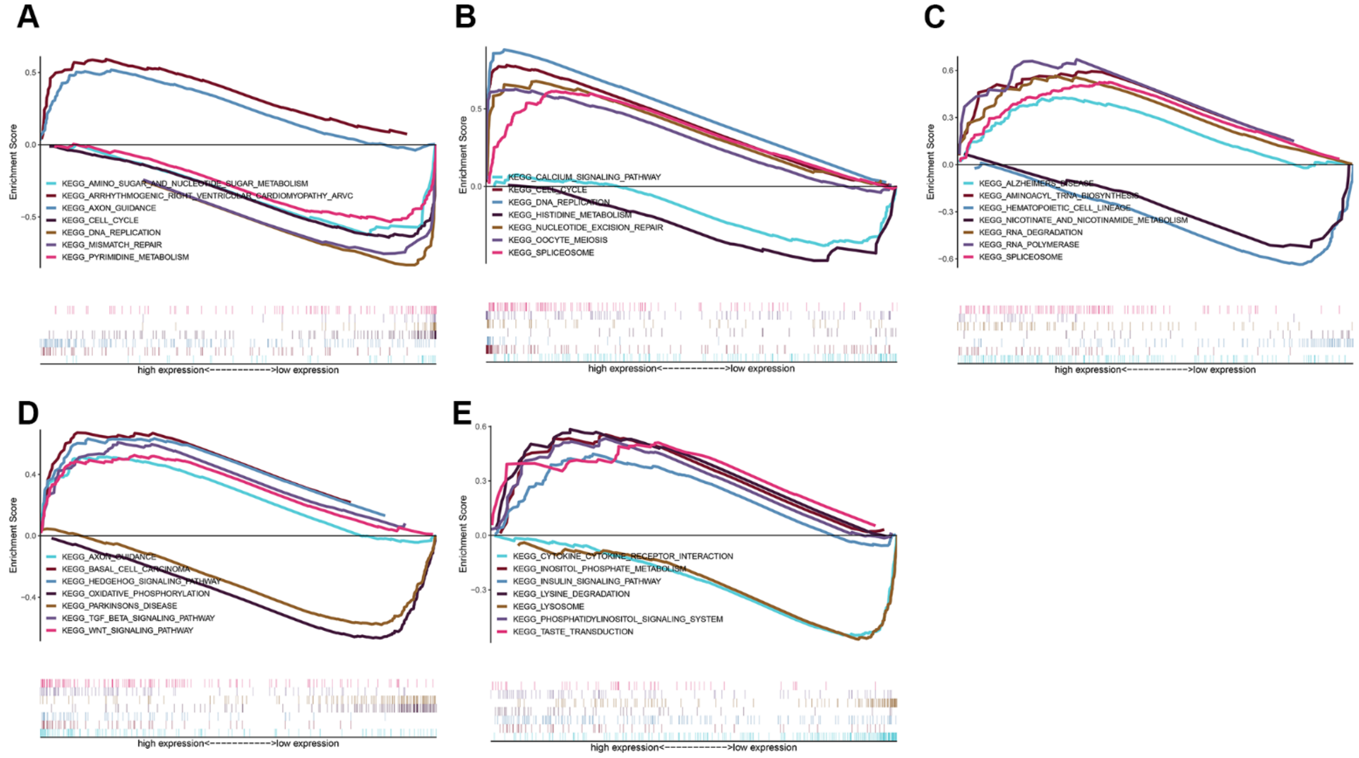

Supplementary Figure 8. Gene Set Enrichment Analysis (GSEA) of the five key genes. GSEA for TSPAN7 (**A**), MYBL2 (**B**), GCSH (**C**), FBN2 (**D**), and DDX39B (**E**).

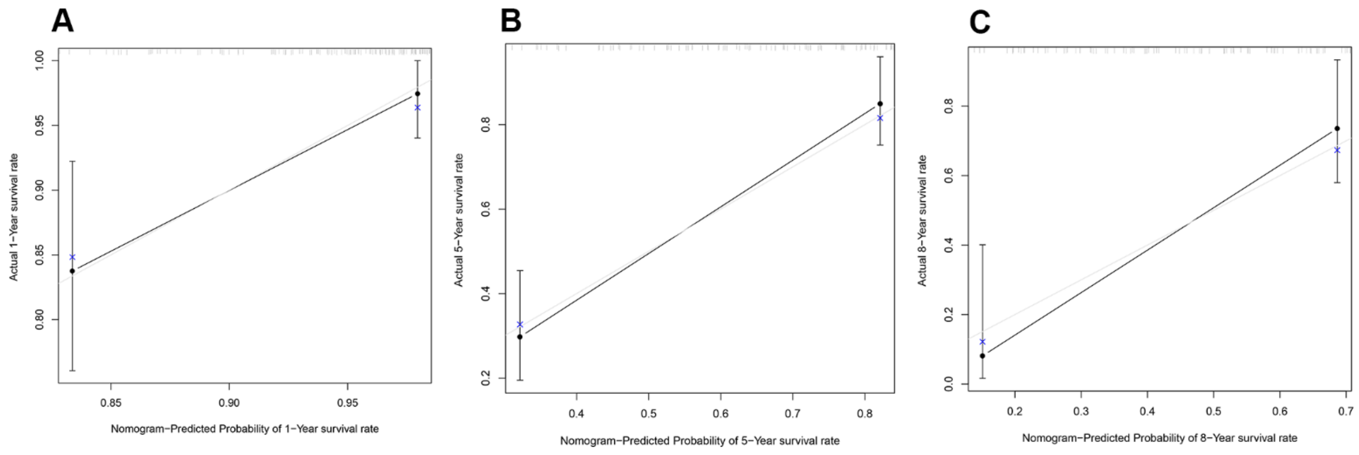

Supplementary Figure 9. Accordance between actual survival rates and prediction of the nomogram.
